# Presumed Autoimmune Keratitis in Both Eyes Without Systemic Manifestations: A 40-Year Course of a Patient With Corneal Infiltrates and Melt

**DOI:** 10.7759/cureus.79852

**Published:** 2025-02-28

**Authors:** Toshihiko Matsuo, Takehiro Tanaka

**Affiliations:** 1 Department of Ophthalmology, Graduate School of Interdisciplinary Science and Engineering in Health Systems, Okayama University, Okayama, JPN; 2 Department of Ophthalmology, Okayama University Hospital, Okayama, JPN; 3 Department of Pathology, Graduate School of Medicine, Dentistry, and Pharmaceutical Sciences, Okayama University, Okayama, JPN

**Keywords:** autoimmune keratitis, corneal graft, corneal infiltration, corneal melt, penetrating keratoplasty

## Abstract

Peripheral corneal infiltration, corneal ulcer, and melt are recognized complications linked to systemic immunological diseases, such as antineutrophil cytoplasmic antibody (ANCA)-associated vasculitis. These manifestations, which occur in isolation, might be autoimmune keratitis but are difficult to prove underlying immunological abnormalities. This report described a patient with presumed autoimmune keratitis who repeatedly presented corneal infiltration and perforation in both eyes even after penetrating keratoplasty. The 68-year-old patient with a stable condition of keratoconjunctivitis sicca, in a 28-year follow-up, abruptly developed mild infiltrates in the corneal center of the right eye and white dense infiltrates in the peripheral and central cornea of the left eye. He was treated with topical 0.1% betamethasone eye drops and oral prednisolone tapering from 30 mg daily. The patient underwent cataract surgeries in both eyes 10 months after the onset of corneal infiltration and subsequently underwent penetrating keratoplasty in both eyes due to abrupt corneal perforation in the left eye 14 months after the onset of corneal infiltration. Six months post-keratoplasty, he experienced a recurrence of infiltrates in the corneal grafts in both eyes, leading to corneal leukoma in the left eye. The corneal graft in the right eye maintained its integrity with relatively mild opacity until approximately 3.5 years post-keratoplasty, when he abruptly developed white dense infiltration of both the corneal graft and his own peripheral cornea at the age of 73. In response to oral prednisolone tapered from 15 mg daily, the corneal infiltration in the right eye resolved but resulted in graft failure. Since he did not exhibit systemic symptoms and signs throughout the course, the repeat episodes of infiltration in both his own cornea and the corneal graft would be the manifestations of autoimmune keratitis. The entity of autoimmune keratitis in isolation would be beneficial to establish a therapeutic strategy for long-term immunosuppression in light of a risk for steroid side effects and a high rate of corneal graft failure.

## Introduction

Non-traumatic corneal melt and perforation is an ophthalmic emergency, and the etiologies should be considered to initiate an appropriate therapy [1,2]. In the list of local ocular surface diseases, corneal thinning as the consequence of keratoconus [3], herpetic stromal keratitis [4], atopic keratoconjunctivitis [5], and keratoconjunctivitis sicca [6] may lead to corneal melt and perforation. In the list of systemic diseases, immunological abnormalities in both non-infectious and infectious diseases may serve as the background for developing non-traumatic corneal melt and perforation. As non-infectious causes, systemic autoimmune diseases such as rheumatoid arthritis and antineutrophil cytoplasmic antibody (ANCA)-associated vasculitis [7] may abruptly develop sterile corneal melt and perforation, either in isolation or in association with scleritis [8] and peripheral corneal infiltration [9,10]. As infectious causes, rare cases of corneal perforation with no direct involvement of infection on the ocular surface have been described to occur in systemic infectious diseases such as syphilis [11], tuberculosis [12], and human immunodeficiency virus infection [13].

Altogether, sterile corneal infiltration, melt, and perforation, which occur in systemic immunological abnormalities, suggest an autoimmune mechanism for an abrupt onset of the corneal melt. Hence, they might be the manifestations of autoimmune keratitis. The potential immunological pathways involved in autoimmune mechanisms, which could be induced by infection or other causes, are T-cell-mediated inflammation, autoantibodies, and cytokine dysregulation. However, the entity of autoimmune keratitis, which occurs in isolation, would be questioned since it is difficult to prove the autoimmune mechanism for corneal melt. Recently, immune checkpoint inhibitors have been widely used for cancer therapy, and their unique autoimmune disease-like side effects in different organs are summarized as immune-related adverse events. In the field of eye diseases, Vogt-Koyanagi-Harada disease-like manifestations are well-known as an autoimmune reaction to melanocytes in the entity of immune-related adverse events [14]. In this context, rare cases with sterile corneal ulcer and perforation [15,16], as well as peripheral corneal infiltration [17], have been reported following the administration of immune checkpoint inhibitors and designated as autoimmune keratitis.

In this study, we report a patient with no systemic disease who repeatedly developed corneal infiltration and melt in both eyes before and after penetrating keratoplasty in the 40-year follow-up period. The course in this patient suggests that the manifestations would be designated as autoimmune keratitis.

## Case presentation

A 40-year-old man who worked as a technician in the engineering department of a university was diagnosed and followed as having keratoconjunctivitis sicca in both eyes, based on the Schirmer test and infiltration with lymphocytes in minor lacrimal glands by lip mucosal biopsy as the histopathological confirmation. He did not show dry mouth symptoms or joint pains. He did not take any medicine and did not have any past history, including rheumatoid arthritis and other autoimmune diseases. He did not smoke or drink alcohol. No abnormalities were detected by annual health checkups at the worksite, which included blood pressure measurements, plain chest X-rays, electrocardiograms, urinalysis, and blood examinations. The best-corrected visual acuity in decimals was 1.2 in both eyes, and the intraocular pressure was 10 mmHg in both eyes. At every three-month visit, slit-lamp examinations showed normal eyelid margins and superficial punctate keratopathy at a constant level in the entire cornea. He showed clear ocular media and a normal fundus in both eyes. He used ionic solution eye drops daily. In case of exacerbation, he additionally used 0.1% fluorometholone eye drops or 0.1% betamethasone eye drops or ointment until remission.

**Figure 1:**
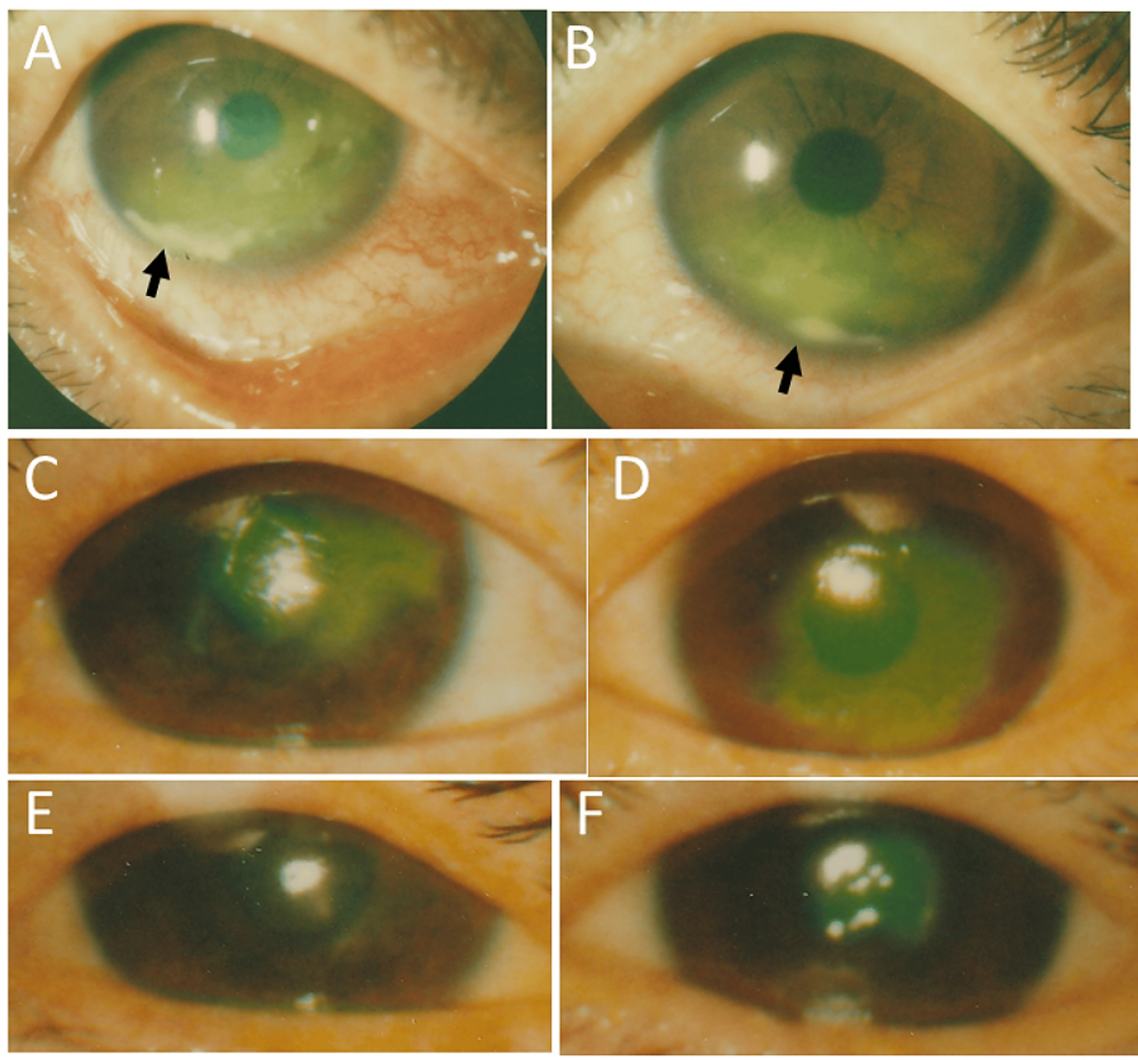
Slit-lamp photomicrographs at the age of 51–52 years Peripheral corneal infiltration (arrows) and decompensated corneal epithelium stained with fluorescein dye in the lower part with bulbar conjunctival injection in both eyes (A: right eye, B: left eye) at the age of 51. Central corneal erosion stained with fluorescein dye in both eyes (C: right eye, D: left eye) eight months later. Corneal erosion in smaller size one week later in both eyes (E: right eye, F: left eye) in response to topical 0.1% betamethasone eye drops.

He was stable until the age of 51 years, when he developed peripheral corneal infiltration and decompensated corneal epithelium stained with fluorescein dye in the lower part with bulbar conjunctival injection in both eyes (Figures 1A, 1B). Topical instillation was switched from 0.1% fluorometholone to 0.1% betamethasone, and the peripheral corneal infiltration in both eyes subsided in a month. Eight months later, he then developed central corneal erosion stained with fluorescein dye in both eyes (Figures 1C, 1D), and the corneal erosion diminished in size one week later in both eyes following the reuse of topical 0.1% betamethasone instead of 0.1% fluorometholone (Figures 1E, 1F).

**Figure 2:**
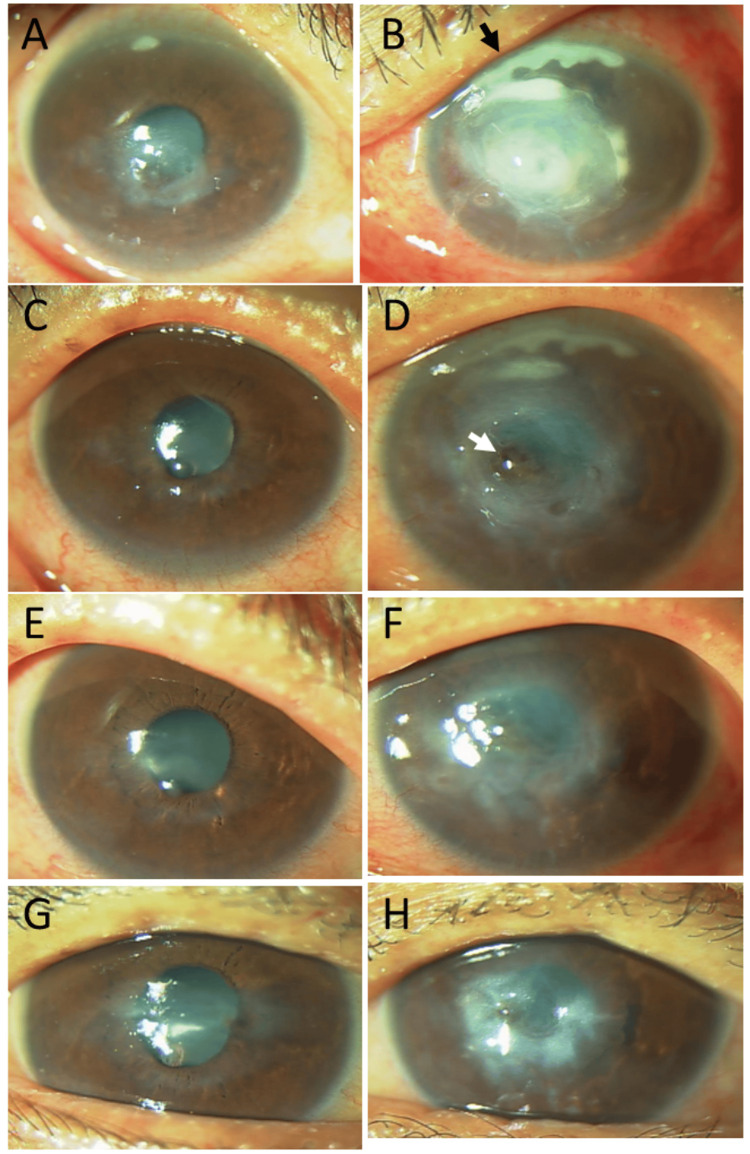
Slit-lamp photomicrographs at the age of 68 years Central corneal infiltration in the right eye (A) and upper peripheral and central corneal dense infiltration in the left eye (B) at the age of 68, 17 years from the initial visit. Clearing of corneal infiltrates one week later (C: right eye, D: left eye) and four weeks later (E: right eye, E: left eye) in response to topical 0.1% betamethasone eye drops, oral prednisolone 30 mg daily, and tapering. Note corneal perforation (arrow) sealed with iris in the left eye (D, F). Irregularly thinned cornea in the right eye (G) and residual corneal opacity with small iris incarceration in the left eye (H) three months later when oral prednisolone was discontinued.

He was stable with topical 0.02% fluorometholone and ionic solution eye drops for 17 years until the age of 68, when he abruptly showed central corneal infiltration in the right eye and upper peripheral and central corneal dense infiltration in the left eye (Figures 2A, 2B). The best-corrected visual acuity in decimals was 0.6 in the right eye and hand movement in the left eye. Corneal sensitivity tests with a cotton thread were normal in both eyes. He had no systemic symptoms or signs, such as fever, skin rashes, and arthralgia. Physical examinations revealed no significant findings. Blood examinations (Table 1) showed normal ranges of complete blood cell counts and blood chemistry tests, except for a mild increase of serum C-reactive protein (CRP) at 1.66 mg/dL (normal range, 0.0-0.30 mg/dL). Serum rheumatoid factor was moderately elevated at 183.6 IU/L (normal range, 0.0-16.0 IU/L), while antinuclear antibody, Sjögren's syndrome-A and B autoantibodies (SS-A and SS-B), and myeloperoxidase or proteinase 3 antineutrophil cytoplasmic antibodies (MPO-ANCA and PR3-ANCA) were all negative. Blood activity of angiotensin-converting enzyme (ACE) was not elevated at 7.3 U/L (normal range, 8.3-21.4 U/L). Serological tests for syphilis, as well as screening tests for hepatitis B virus antigen, hepatitis C virus antibody, and human immunodeficiency virus antigen/antibody, were all negative. Urinalysis was normal. A plain chest X-ray disclosed no abnormalities. The corneal lesion in the left eye swabbed for Gram staining and culture was negative for microorganisms, and the conjunctival sac culture in both eyes was negative for bacteria.

**Table 1:** Blood examinations at the age of 68 years with abrupt corneal infiltration in both eyes and at the age of 74 years with residual corneal leukoma in both eyes Normal ranges at the in-house laboratory were for the time at 68 years. All values are reported in standard units. n.d.: not determined

Parameters	Normal Range	At 68 Years	At 74 Years
Red blood cells (× 10^6^/µL)	4.30-5.70	4.02	4.09
Platelets (× 10^3^/µL)	150-350	259	212
White blood cells (× 10^3^/µL)	3.50-8.50	5.96	6.05
Neutrophils (%)	35.0-73.0	61.9	70.7
Lymphocytes (%)	20.0-52.0	26.8	17.3
Monocytes (%)	0.0-13.0	7.0	8.5
Eosinophils (%)	0.0-11.0	3.7	2.5
Basophils (%)	0.0-2.0	0.6	0.9
Hemoglobin (g/dL)	13.5-17.0	14.3	14.7
Hematocrit (%)	40.0-50.0	41.9	41.9
Total protein (g/dL)	6.5-8.0	8.3	6.9
Albumin (g/dL)	3.9-4.9	4.5	4.1
Lactate dehydrogenase (LD) (U/L)	120-240	215	204
Aspartate aminotransferase (AST) (U/L)	10-35	28	25
Alanine aminotransferase (ALT) (U/L)	7-42	27	27
γ-glutamyl transferase (γ-GT) (U/L)	5-60	55	46
Creatine kinase (CK) (U/L)	41-258	134	88
Total bilirubin (mg/dL)	0.33-1.28	0.57	0.74
Urea nitrogen (mg/dL)	8.1-22.0	15.0	9.9
Creatinine (mg/dL)	0.60-1.10	0.60	0.69
Estimated glomerular filtration rate (eGFR) (mL/minute/1.73 m^2^)	60 or greater	101.1	84.7
Uric acid (mg/dL)	3.5-7.0	5.5	6.0
Total cholesterol (mg/dL)	130-220	252	249
Postprandial blood glucose (mg/dL)	<140	119	120
C-reactive protein (CRP) (mg/dL)	0.00-0.30	1.66	n.d.
Sodium	136-144	139	141
Potassium	3.7-4.9	5.0	4.7
Chloride	102-110	103	108

He began to have topical 0.1% betamethasone eye drops and oral prednisolone 30 mg daily, which was tapered and discontinued in three months since he complained of muscle pain in the lower limbs. In response to prednisolone, the corneal infiltrates in both eyes cleared in a week but ended up with irregular corneal thinning in the right eye (Figure 2C) and central corneal perforation with iris incarceration in the left eye (Figure 2D). After three months of prednisolone treatment, the cornea of the right eye exhibited thinning and an irregular shape (Figures 2E, 2G), while the left eye showed residual central corneal opacity with a small area of iris incarceration (Figures 2F, 2H). No additional measures were taken in the left eye, which maintained the integrity of the anterior chamber by the healing incarceration. Half a year later, he developed a posterior subcapsular cataract in the right eye and a mature cataract in the left eye. He underwent cataract surgery with intraocular lens implantation in the right eye, while the left eye received cataract surgery without intraocular lens implantation since the lens capsule was too weak in the limited visibility during the surgery. The visual acuity in decimals was 0.2 in the right eye and 0.03 in the left eye after the cataract surgeries.

**Figure 3:**
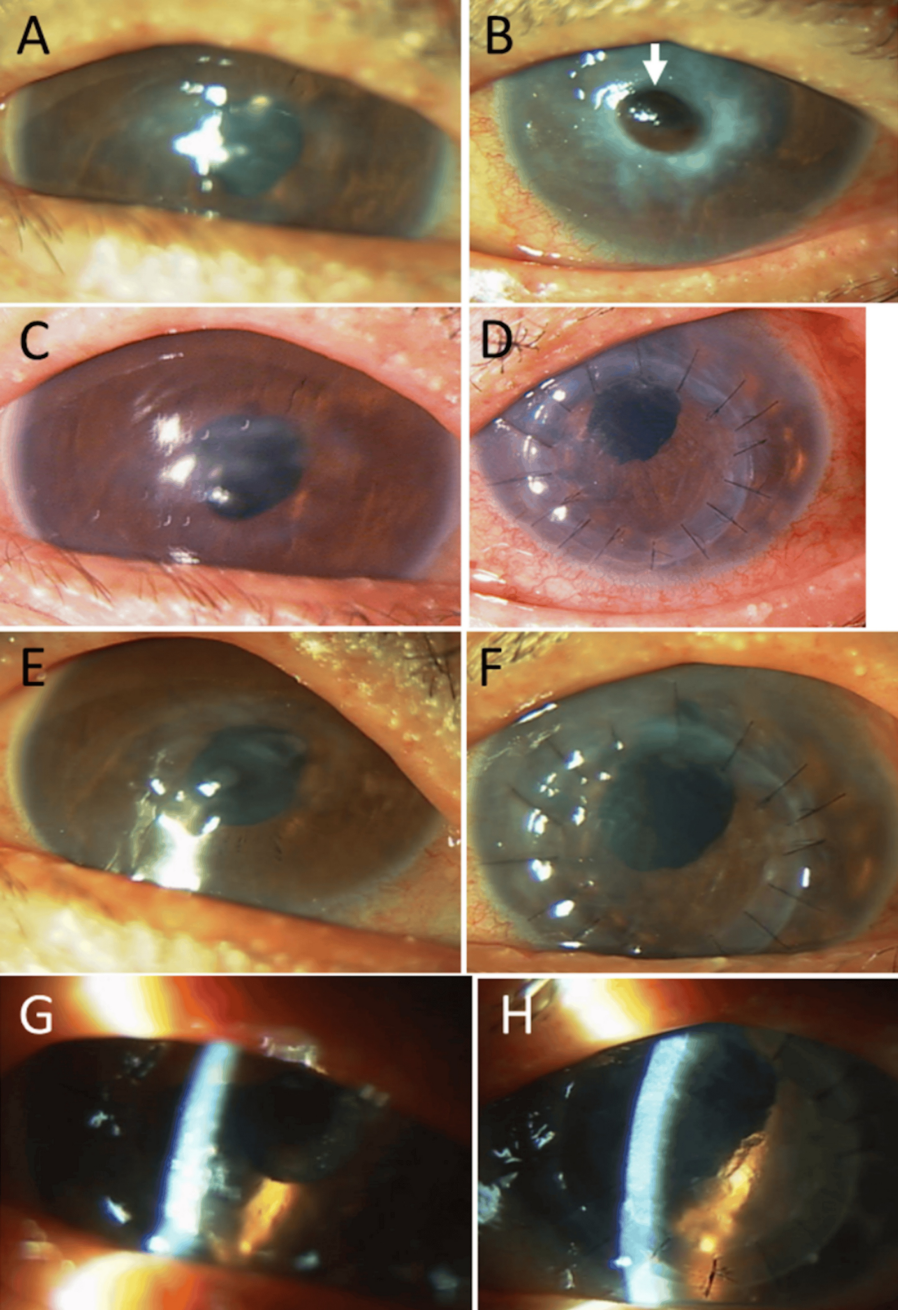
Slit-lamp photomicrographs at the age of 69–70 years A stable thinned cornea in the right eye (A) and abrupt corneal perforation with iris incarceration in the left eye (B) at the age of 69 years, 3.5 months after cataract surgeries in both eyes and 14 months after the episode of corneal infiltration. A stable thinned cornea in the right eye (C) and a clear corneal graft in the left eye (D) one week after emergency keratoplasty with intraocular lens suturing. A stable thinned cornea in the right eye (E) and a clear corneal graft in the left eye (F) one month after keratoplasty. A clear corneal graft in the right eye (G) one month after keratoplasty and a clear corneal graft in the left eye (H) three months after keratoplasty.

**Figure 4:**
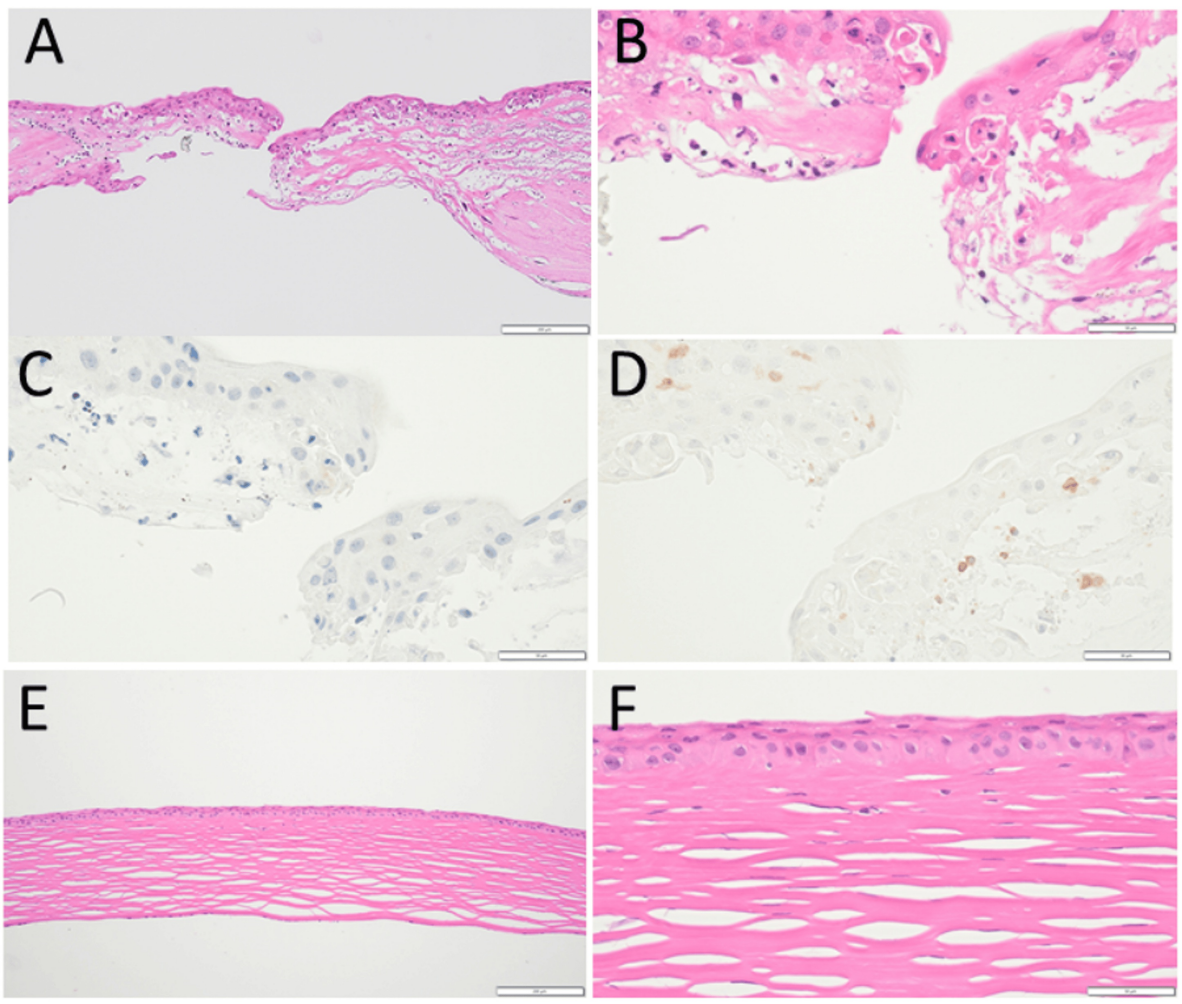
Pathological photomicrographs Pathological examinations of the perforated cornea in the left eye (A-D) extirpated at penetrating keratoplasty at the age of 69 years and the extirpated cornea in the right eye (E, F) at penetrating keratoplasty two months later. Note CD20-negative (C) and CD3-positive (D) T-cells at the edge of corneal perforation. No inflammatory cells in the cornea of the right eye (E, F). Hematoxylin & eosin stain in A, B, E, and F. White scale bar = 200 µm in A and E; bar = 50 µm in B, C, D, and F.

At the age of 69, he developed central corneal perforation with iris incarceration in the left eye (Figure 3B), 3.5 months after cataract surgeries in both eyes and 14 months after the episode of corneal infiltration. The right eye showed a thin cornea with an irregular shape in the stable condition (Figures 3A, 3C, 3E). One week later, he underwent penetrating keratoplasty with intraocular lens suturing in the left eye (Figures 3D, 3F, 3H). Two months later, he also underwent penetrating keratoplasty in the right eye (Figure 3G). The visual acuity in decimals was 0.06 in the right eye and 0.02 in the left eye. Oral prednisone 15 mg daily was given for two weeks after keratoplasty in each eye. Pathological examinations of the perforated cornea extirpated at keratoplasty in the left eye showed infiltration at the edge of the perforation with inflammatory cells (Figures 4A, 4B), which were CD20-negative (Figure 4C) and CD3-positive (Figure 4D) T-cells. At keratoplasty two months later, the extirpated cornea in the right eye showed no inflammatory cells (Figures 4E, 4F).

**Figure 5:**
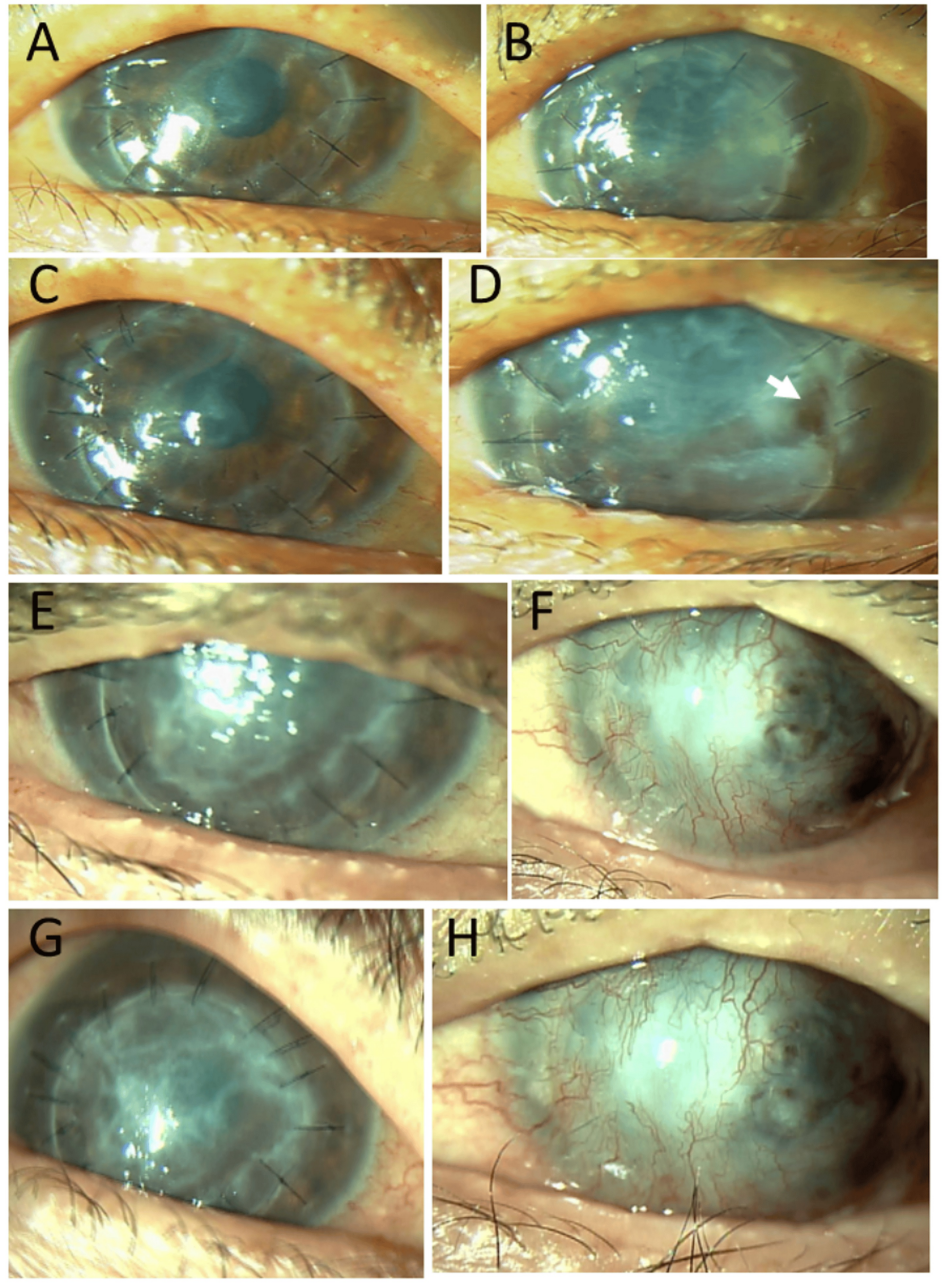
Slit-lamp photomicrographs at the age of 70–71 years Relapsed infiltration of corneal grafts in both eyes (A: right eye, B: left eye) at the age of 70 years, three months after keratoplasty in the right eye and five months after keratoplasty in the left eye. Stable cornea in the right eye (C) and corneal thinning with iris incarceration (arrow) in the left eye (D) one month later, in response to oral prednisolone tapered from 15 mg. Corneal graft with residual infiltration in the right eye (E, G) and corneal leukoma with vascularization in the left eye (F, H) at the age of 71 years, 15 months later and 19 months later, respectively, from infiltration relapse.

He maintained clear corneal grafts in the right eye (Figure 3G) three months after keratoplasty and in the left eye (Figure 3H) five months after keratoplasty until the age of 70 years, when he again developed infiltration of the corneal grafts in both eyes (Figures 5A, 5B). He resumed oral prednisolone 15 mg daily, which was tapered and discontinued in a month due to muscle pain in the lower limbs. The corneal graft in the right eye was relatively transparent (Figure 5C), while the corneal graft in the left eye remained hazy with peripheral iris incarceration (Figure 5D). One year later, at the age of 71 years, he developed again the infiltration of the corneal graft in the right eye (Figure 5E). He showed corneal leukoma with vascularization in the left eye (Figures 5F, 5H) 15 months following the previous corneal infiltration. At that time, he used topical 0.1% betamethasone and did not take oral prednisolone based on his wish.

**Figure 6:**
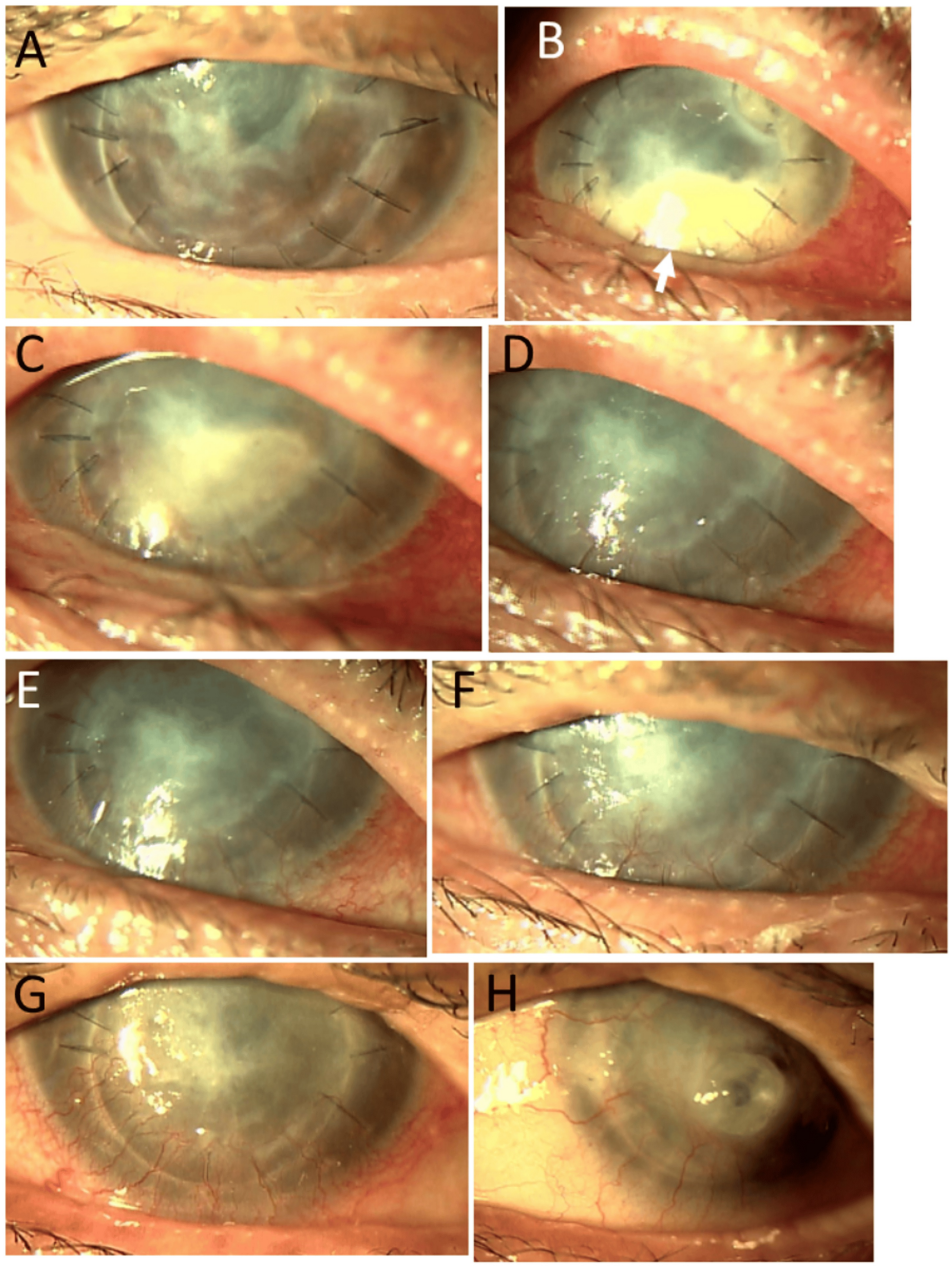
Slit-lamp photomicrographs at the age of 73–76 years Stable residual corneal infiltrate in the right eye (A) at the age of 72, four months after the photo in Figure 5G. Marked infiltrates from the lower peripheral cornea into the corneal graft in the right eye (B) at the age of 73, 17 months subsequent to (A). The corneal infiltrates subsiding with oral prednisolone 15 mg daily and tapering one week later (C), four weeks later (D), six weeks later (E), and eight weeks later (F), finally with corneal scarring. Corneal leukoma in both eyes (G: right eye, H: left eye) at the age of 76, three years from corneal infiltrate relapse in the right eye.

The corneal graft in the right eye remained relatively stable for a year (Figures 5G, 6A) until the age of 73 years, when he abruptly developed marked infiltrates from the lower peripheral cornea into the corneal graft in the right eye (Figure 6B). The corneal infiltrates in the right eye subsided with topical 0.1% betamethasone eye drops and oral prednisolone 15 mg daily, which was tapered and discontinued in a month (Figures 6C-E). The corneal graft in the right eye developed scarring (Figure 6F) two months after the relapse of infiltration. At that time, blood examinations indicated no significant findings (Table 1). At the age of 76, three years after corneal infiltrate relapse in the right eye, the visual acuity was hand movement in the right eye and light perception in the left eye (Figures 6G, 6H). He only used hyaluronate eye drops and no longer used corticosteroid eye drops. He suffered from muscle weakness of the right upper limb caused by brain infarction at the age of 76. One year later, at 77, he underwent coronary artery stent insertion and catheter ablation for atrial fibrillation. At 78, he was diagnosed with parkinsonism and commenced treatment with a levodopa/carbidopa combination, in addition to clopidogrel 75 mg, rivaroxaban 15 mg, and bepridil 50 mg daily for coronary stent and atrial fibrillation, cilnidipine 5 mg for hypertension, and rosuvastatin 2.5 mg daily for dyslipidemia. He maintained the same level of visual acuity until the final follow-up at age 80, when he was confined to a nursing home due to cognitive decline.

## Discussion

The present patient experienced repeat infiltration of his own cornea and the corneal grafts in both eyes after penetrating keratoplasty. The infiltration resulted in corneal thinning and perforation in the left eye. He did not have any systemic diseases that predisposed him to corneal infiltration and melt. Keratoconjunctivitis sicca would serve as one of the precipitating factors for the corneal melt in this patient, but the preceding corneal infiltration in the patient could not be explained by keratoconjunctivitis sicca. Instead, peripheral corneal infiltration in the initial episode, which appeared to advance to the corneal center in the left eye, is similar to the clinical features of ANCA-associated vasculitis [7].

In the same pattern, peripheral corneal infiltration in the third episode advanced, this time to the corneal graft in the right eye. In the course of the eye disease, the patient did not show any systemic symptoms or signs. He only showed a moderately elevated serum level of rheumatoid factor but did not have autoantibodies such as ANCA. The physical examinations and the blood tests were performed in the course to rule out systemic autoimmune diseases such as rheumatoid arthritis and ANCA-associated vasculitis, as well as infectious causes such as syphilis, tuberculosis, and human immunodeficiency virus infection. Under the circumstances, the episodes suggest that an autoimmune mechanism of an unknown entity may underlie the corneal manifestations. Pathological examinations for the tissue obtained at keratoplasty showed infiltration with T-cells but not with neutrophils at the margin of the corneal perforation in the left eye, supporting the presence of immunological abnormalities. In contrast, the thinned cornea in the right eye, which was excised at keratoplasty, showed no inflammatory cells, indicative of the subsidence of inflammation by topical corticosteroids. Unfortunately, in the present patient, blood cytokines, which might provide an additional key for understanding the immunological status, were not measured since the measurements were not the standard at that time.

In the differential diagnosis, the absence of mutton-fat keratic precipitates in the present patient did not support the diagnosis of herpetic stromal or endothelial keratitis. Mutton-fat keratic precipitates are usually observed in corneal graft failure, and dense infiltration of the peripheral recipient cornea, together with infiltration of the corneal graft, is not typical for corneal graft failure. Corneal infection with bacteria and fungi was also denied by the negative culture. Good response to oral prednisolone alone also supported non-infectious causes for corneal infiltration in the present patient. The patient did not use eye drops of non-steroidal anti-inflammatory drugs (NSAIDs), which have been known to develop sterile corneal ulcers and melt, especially in patients with rheumatoid arthritis and other collagen vascular diseases [18,19].

In the literature, corneal infiltration, ulcer, and melt have been described in patients with cancers in the course of immune checkpoint inhibitor administration [15-17]. In the administration of atezolizumab, an anti-PD-L1 antibody, one patient with metastatic bladder cancer developed a corneal ulcer in both eyes [15], while another patient with unresectable hepatocellular carcinoma developed corneal perforation in the left eye only [16]. In contrast, one patient with advanced small-cell lung cancer developed peripheral corneal infiltration in both eyes in the course of atezolizumab [17]. It is intriguing that all three patients were undergoing treatment with atezolizumab. These non-infectious corneal manifestations have been designated as autoimmune keratitis in the entity of immune-related adverse events.

In a different line of interpretation, dry eye condition caused by lacrimal gland inflammation in Sjögren syndrome or keratoconjunctivitis sicca as immune-related adverse events of immune checkpoint inhibitors might be a precipitating factor to develop corneal ulcer [20]. However, peripheral corneal infiltration in the previous patient in the course of atezolizumab [17] could not be attributed to a dry eye condition. In the same way as that patient, corneal infiltration in the present patient could not be attributed to a dry eye condition and might be designated as autoimmune keratitis.

The present patient maintained ambulatory vision for a while after prednisolone treatment for massive infiltration of the corneal graft and his own peripheral cornea in the right eye at the age of 73. A second round of keratoplasty was recommended as a therapeutic option in the present patient after the corneal graft failure in the right eye. In the meantime, the patient had brain infarction and heart disease to cope with at first and did not have a chance to have keratoplasty. In the 40-year course of follow-up, he did not show any signs of systemic autoimmune diseases, and presumed autoimmune keratitis in both eyes appeared to occur in isolation. The presumed autoimmune keratitis in the patient showed a good response to oral corticosteroids. Since the patient complained of muscle pain in the lower extremity in the initial course of oral prednisolone 30 mg daily, repeat events of the presumed autoimmune keratitis were managed with oral prednisolone tapered from 20 mg or 15 mg daily. In retrospect, immunosuppressive agents such as cyclosporin and methotrexate in long-term administration might be considered as therapeutic regimens.

## Conclusions

The 68-year-old patient with a stable condition of keratoconjunctivitis sicca in a 28-year follow-up abruptly developed mild infiltrates in the corneal center of the right eye and white dense infiltrates in the peripheral and central cornea of the left eye, which responded to oral prednisolone tapering from 30 mg daily. In one and a half years, he developed corneal thinning in the right eye and abrupt corneal perforation in the left eye. He underwent penetrating keratoplasty in both eyes but developed again infiltrates in the corneal grafts in both eyes, leading to corneal leukoma in the left eye. At 73 years old, he abruptly developed white dense infiltration of the corneal graft and his own peripheral cornea in the right eye, leading to graft failure. Since he did not have systemic symptoms and signs throughout the course, the repeat episodes of infiltration in his own cornea and also in the corneal graft would be the manifestations of autoimmune keratitis. The entity of autoimmune keratitis in isolation would be supported by similar manifestations during immune checkpoint inhibitor administration.

Autoimmune keratitis should be considered in the differential diagnoses of corneal infiltration and melt. The course of the present patient suggests that it would be desirable to establish a therapeutic strategy for long-term immunosuppression in light of the risk of steroid side effects and a high rate of corneal graft failure.
